# Factors influencing Chinese young adults' intention to receive HPV vaccination: the mediating role of attitude

**DOI:** 10.3389/fpubh.2025.1612480

**Published:** 2025-07-23

**Authors:** Hongyan Wu, Guateng Liow, Zhengxi Zhou, Jifeng Li

**Affiliations:** ^1^School of Humanities and Management, Guilin Medical University, Guilin, China; ^2^School of Business, Shandong Xiehe University, Shandong, China; ^3^Finance Department, Guilin Medical University, Guilin, China

**Keywords:** HPV vaccination, health belief model, Theory of Planned Behavior, behavioral intention, attitude, Chinese young adults

## Abstract

**Introduction:**

This study examines the intention of young adults to receive the Human Papillomavirus vaccination in China, with a specific focus on the mediating role of attitude within an integrated framework of the Health Belief Model and the Theory of Planned Behavior.

**Methods:**

A convenience sampling method was utilized to collect data through an online questionnaire targeting young adults aged 18 to 26. The data were analyzed using Partial Least Squares Structural Equation modeling to explore the relationships between various factors affecting vaccination intention.

**Results:**

The findings revealed that perceived susceptibility, perceived severity and perceived benefits have significant positive effects on attitudes toward the HPV vaccine. Subjective norms, attitude and perceived behavioral control positively influence the intention to receive the HPV vaccination. Attitude was identified as a significant mediator between these variables and intention to receive HPV vaccination.

**Discussion:**

This study highlights the need for targeted educational campaigns to improve young adults' HPV vaccine attitudes. Public health initiatives may potentially increase HPV vaccination rates.

## Introduction

Human papillomavirus (HPV) is a globally prevalent sexually transmitted infection, affecting both men and women. According to the Centers for Disease Control and Prevention, nearly all sexually active individuals will contract HPV at some stage in their lives (1, 2). In China, HPV poses a significant public health burdens, with an estimated infection rate of 17.70% (3) and a substantial burden of HPV-related cancers, especially cervical cancer (4). It underscores the critical need for effective vaccination strategies to mitigate the impact of HPV on public health.

Vaccine hesitancy has become a pressing global health concern, the World Health Organization listed it as one of the top ten public health threats in 2019 (5). In China, HPV vaccination rates among young adults remain alarmingly low. A nationwide survey of 4,220 students revealed that only 11.0% had received the HPV vaccine (6). Similarly, another study involving 4,000 females and 1,000 males across 31 provinces found that a mere 3% of female participants had been vaccinated (7). These findings underscore the urgent need to identify and address barriers to HPV vaccine uptake among young adults.

To better understand vaccine acceptance, researchers have applied various theoretical frameworks, including the Health Belief Model (HBM) (8), Protection Motivation Theory (9), Theory of Planned Behavior (TPB) (10), Social Cognitive Theory (11), and the Transtheoretical Model (12). However, no single theory has consistently demonstrated superior predictive power in explaining HPV vaccination behavior (13–19). This study proposed an integrated model that combines perceived susceptibility, perceived severity and perceived benefits with TPB to understand HPV vaccination intention among Chinese young adults. This integrated model aims to enhance the model's predictive validity and providing deeper insights into the psychological determinants of vaccination decisions. The findings could inform targeted interventions to increase HPV vaccine acceptance among Chinese young adults. Specifically, this study focuses on the mediating role of attitude, understanding how attitude mediates the relationship between perceived threat and benefits and vaccination intentions can provide valuable insights for developing effective public health campaigns.

## Theoretical background

### Health belief model

The HBM is a well-established theoretical framework grounded in cognitive psychology, employed to examine health-related decision-making and health behavior adoption. The model posits that individuals' health behaviors are shaped by their perceived susceptibility and severity of a health threat, as well as by their assessment of the benefits and barriers of preventive actions (20). Additionally, the HBM incorporates cues to action that prompt behavioral responses and self-efficacy, which indicates an individual's confidence in their ability to carry out the desired behavior. The HBM is recognized as a dominant framework for health behavior research (21). Empirical studies have extensively applied the HBM across various health domains, providing a solid foundation for health intervention design, including breast cancer screening (22, 23), weight management (24), self-care practices (25), cervical cancer screening (26), oral health behaviors (27), and vaccination adherence (28). However, evidence indicates that HBM's predictive power is relatively weak (29). It suggests that theoretical integration is needed to enhance its explanatory power.

### Theory of Planned Behavior

TPB, articulated by Ajzen (10) has been instrumental in explaining a broad spectrum of behaviors, notably in the realm of health. At the core of this theoretical model lies behavioral intention, which represents the most immediate and powerful determinant of actual behavior, capturing an individual's motivation and commitment to perform a specific action. This theory posits that behavioral intentions are determined through an interplay of three core psychological factors. Attitude toward the behavior represents an individual's overall favorable or unfavorable assessment of performing the specific action. Subjective norms capture the perceived social expectations from important referents and the motivation to comply with these social pressures. Perceived behavioral control incorporates the perceived obstacles or facilitators to performing the behavior and the person's self-efficacy in overcoming potential barriers (10). These factors interact dynamically to influence an individual's likelihood of acting on their intentions, thus playing a critical role in predicting and understanding behavioral change. TPB is esteemed as a suitable theoretical framework due to its robustness and simplicity, demonstrating wide-ranging predictive power for human actions. It has been corroborated in over 1,000 empirical studies, including those examining sex behaviors (30), breast self-examination practices (31).

### Integrating the health belief model and Theory of Planned Behavior

**Figure 1:**
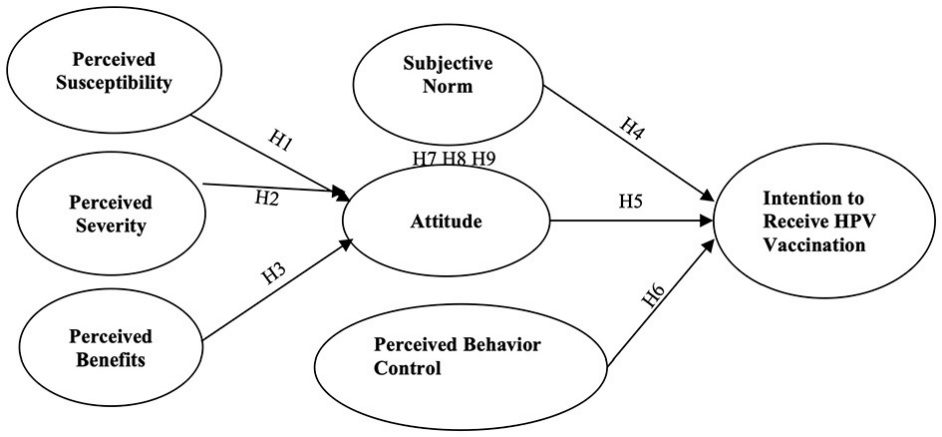
Research model.

This study integrated HBM and TPB into a unified theoretical framework to improve and predict behavioral intentions toward HPV vaccination (see Figure 1). Existing research has applied these theories independently, but their combined potential to explain behavioral variance remains underexplored. Drawing on Baranowski's (32) assertion that integrating robust predictors enhances the explanatory power of health behavior models, this study addresses critical overlaps and gaps between HBM and TPB. Notably, constructs such as HBM's perceived barriers and self-efficacy exhibit conceptual alignment with TPB's perceived behavioral control (33). To mitigate multicollinearity risks, these overlapping constructs (i.e., barriers and self-efficacy) are excluded from the integrated model, preserving theoretical distinctiveness. Prior studies comparing the HBM and TPB have predominantly focused on their predictive utility rather than synthesizing their frameworks to elucidate causal pathways (34). This integration is critical, as TPB does not explicitly account for health-specific threat and benefit appraisals central to the HBM. Fishbein and Yzer (35) argue that threat-benefit analyses should inform attitude formation within TPB. These constructs reflect behavioral beliefs, which act as antecedents to attitudes and intentions (36–39). This study examines the interplay between health cognitions and TPB core determinants by unifying these constructs. This approach advances theoretical understanding by delineating how health beliefs catalyze attitude formation and subsequent intention development. This study thus provides a nuanced lens to explore HPV vaccination bridging health-specific cognitions and social-behavioral mechanisms.

## Research model and hypotheses

### Perceived susceptibility

Perceived susceptibility involves an individual's subjective evaluation of their likelihood of acquiring a specific disease or health-related condition (20). Rooted in the HBM (8), this construct posits that heightened perceptions of vulnerability to adverse health outcomes motivate individuals to adopt protective behaviors. Empirical studies indicate substantial variability in perceived susceptibility across populations, with elevated risk perceptions consistently linked to greater engagement in preventive actions (40). For instance, Liao et al. (41) demonstrated a significant positive association between perceived susceptibility and affective attitude in the context of influenza vaccination. Extending these findings to HPV vaccination behaviors, the following hypothesis is put forward:

H1: Perceived susceptibility will positively influence young adults' attitudes toward HPV vaccine.

### Perceived severity

Perceived severity involves an individual's subjective assessment of the potential harm or adverse consequences related to a health-related threat (20). This construct is commonly operationalized through evaluations of the clinical and psychosocial seriousness of a disease or health condition, encompassing both physical outcomes (e.g., pain, disability, mortality) and socio-functional ramifications (e.g., occupational disruption, familial strain) (40, 42). Empirical investigations, such as Bulgurcu et al. (43), demonstrate that heightened threat salience correlates with enhanced motivation to adopt protective behaviors through attitude formation. Recent studies further substantiate this relationship. Chu et al. (44) identified a direct positive association between perceived severity and favorable attitudes toward vaccination, while Lennox et al. (45) highlighted its predictive utility in shaping attitudes toward preventive health technologies. Consequently, the following hypothesis is put forward:

H2: Perceived severity will positively influence young adults' attitudes toward HPV vaccine.

### Perceived benefits

Perceived benefits refer to an individual's evaluation of the potential advantages or effectiveness of adopting health-protective behaviors to reduce disease risk (20). Within HBM, this construct posits that behavioral adoption is driven by the anticipation of tangible outcomes, such as lowered disease susceptibility or avoidance of health complications. In the context of HPV vaccination, perceived benefits may include direct health advantages (e.g., immunity against high-risk HPV strains) and indirect gains, such as reduced long-term healthcare expenditures or enhanced quality of life. The meta-analysis conducted by Janz and Becker (40) demonstrated that in 81% of the studies reviewed, perceived benefits were identified as the second most influential element among the core constructs of the HBM. The scope of perceived benefits extends beyond traditional health contexts. In consumer behavior research, Choi et al. (46) demonstrated that perceived benefits significantly enhance attitudes toward street food consumption. Similarly, Al-Debei et al. (47) revealed that perceived benefits serve as primary drivers of positive attitudes toward online shopping platforms. Recent investigations into health-related behaviors further corroborate this relationship. Yasa et al. (48) discovered that perceived benefits of medical mask usage exerted a positive effect on public attitudes toward mask adoption. According to previous studies, the following hypothesis is put forward:

H3: Perceived benefits will positively influence young adults' attitudes toward HPV vaccine.

### Attitude

Within the TPB, attitude is conceptualized as a central antecedent to behavioral intentions and actions, reflecting an individual's favorable or unfavorable evaluation of engaging in a specific behavior (10). Empirical evidence consistently demonstrates that positive attitudinal evaluations increase the likelihood of adopting health-protective behaviors (49–51). In the context of HPV vaccination, numerous studies underscore the predictive utility of attitude. Catalano et al. (49) identified attitude as the strongest predictor of vaccination intentions among male college students. Similarly, Jozkowski and Geshnizjani (50) reported a robust association between favorable attitudes and vaccination intentions in female college students. Askelson et al. (13) revealed that maternal attitudes significantly predicted intentions to vaccinate adolescent daughters. Collectively, these findings affirm attitude as a critical determinant of HPV vaccination. Hence, the following hypothesis is put forward:

H4: Attitude will positively influence young adults' intention to receive the HPV vaccination.

### Subjective norm

Subjective norm, a core construct of the TPB, is shaped by normative beliefs that reflect an individual's perception of significant others' expectations regarding their engagement in or avoidance of a specific behavior (52). Recent studies, such as that by Prasetyo et al. (53), have demonstrated the significant impact of subjective norms on the intention to comply with preventive health measures. In the context of HPV vaccination, several investigations have underscored the influence of subjective norms on individuals' intention to receive the vaccination. Reiter et al. (54) noted that young males who valued peer approval experienced regret for not being vaccinated against HPV and perceived an increased likelihood of vaccination due to perceived risk. Askelson et al. (13) also confirmed that subjective norms influenced mothers' intentions to vaccinate their daughters against HPV. Drawing on prior research, the following hypothesis is put forward:

H5: Subjective norm will positively influence young adults' intention to receive the HPV vaccination.

### Perceived behavioral control

Perceived behavioral control reflects an individual's confidence in their capability to carry out a particular behavior, which is affected by perceived access to resources, opportunities, and self-efficacy (10). Empirical evidence underscores its predictive validity across health behaviors. A meta-analysis by Cooke and French (55) of 33 studies demonstrated that perceived behavioral control moderately to strongly predicts behavioral intentions, with higher perceived behavioral control correlating with a stronger intention to act. In the context of HPV vaccination, a stronger perceived behavioral control is anticipated to foster a stronger intention to get vaccinated. Numerous studies (13, 56–58) have found that perceived behavioral control has a significant and positive impact on the intention to receive the HPV vaccine. Consequently, the following hypothesis is put forward:

H6: Perceived behavioral control will positively influence young adults' intention to receive the HPV vaccination.

### The mediating role of attitude

Attitude, a core component in Fishbein and Ajzen's (59) belief-attitude-intention framework, is defined by Casalo et al. (60) as a learned predisposition toward positive or negative evaluations of specific objects. In the present study, attitudes toward HPV vaccine are influenced by an individual's health beliefs and environmental factors. This study used the transmittal approach (61) to elucidate the mediating role of young adult's attitudes toward HPV vaccines. Specifically, it is hypothesized that attitude will intervene in relationship between perceived susceptibility, perceived severity, perceived benefits and the intention to receive HPV vaccination. Extensive research has consistently identified attitude as a primary determinant of behavioral intention across diverse contexts (62–64). Haddock and Maio (65) define attitude as an evaluative judgment directed toward a stimulus object, which aligns with the Stimulus-Organism-Response paradigm proposed by Mehrabian and Russell (66). This paradigm delineates how an individual's response to environmental stimuli. Within this study, the stimulus (dimensions of HBM beliefs) impacts attitude (the organism), which in turn influences the intention to receive the HPV vaccination (the response). Empirical evidence from various settings corroborates the mediating role of attitude, which is shaped by health beliefs such as perceived susceptibility, perceived severity, and perceived benefits (43, 67–70). Accordingly, the following hypothesis is put forward:

H7: Attitude toward the HPV vaccine mediates the relationship between perceived susceptibility and intention to receive vaccination.
H8: Attitude toward the HPV vaccine mediates the relationship between perceived severity and intention to receive vaccination.
H9: Attitude toward the HPV vaccine mediates the relationship between perceived benefits and intention to receive vaccination.

## Materials and methods

### Participants

The convenience sampling method was employed due to the absence of a comprehensive sampling frame for young adults aged 18–26 in China. This target population was selected for two main reasons. First, catch-up vaccinations are recommended up to the age of 26 (71). Second, in China, individuals under 18 years of age are legally considered minors, with parents typically exercising decision-making authority regarding their children's vaccinations. In contrast, young adults generally have greater autonomy in deciding whether to receive vaccinations. Therefore, this study targets young adults aged 18 to 26 as survey participants.

### Data collection

The research employed a cross-sectional design utilizing a web-based survey platform hosted on www.wjx.cn, with dissemination channels comprising mainstream Chinese social media applications (WeChat and QQ). The privacy of the participants was strictly safeguarded. Data collection was conducted from March 2 to April 5, 2024, with 581 online questionnaires completed. After data cleaning, 516 valid responses were retained. Table 1 presents the demographic characteristics of the participants, which includes information on sex, age, occupation, educational background, scared of needles.

**Table 1:** The demographics of the sample *(N* = 516).

**Demographic variables**	**Description**	**Frequency**	**Percentage**
Sex	Male	241	46.7
	Female	275	53.3
Age	18–20 years old	190	36.8
	21–23 years old	197	38.2
	24–26 years old	129	25.0
Occupation	Employment	252	48.8
	Unemployed	48	9.3
	Students	216	41.9
Educational background	High school or below	72	14.0
	College degree	259	50.2
	Bachelor's degree	155	30.0
	Master's degree or above	30	5.8
Scared of injections	Not scared	422	81.8
	A little scared	71	13.8
	Very scared	23	4.4

## Measures

### Perceived susceptibility

Perceived susceptibility were assessed with six items, adapted from Champion (20): “My chances of getting the HPV virus are great,” “It is likely that I will be infected with the HPV virus,” “I feel that my chances of getting HPV-related cancer in the future are high,” “There is a good possibility that I will get HPV-related cancer,” “I worry a lot about getting HPV-related cancer,” and “In the future, I may get HPV-related cancer.” These items utilized a 5-point Likert scale (1 = “strongly disagree” to 5 = “strongly agree”).

### Perceived severity

Perceived severity were assessed with six items, adapted from Krawczyk et al. (72): “I think HPV infection is serious,” “If I am infected with HPV, it would be serious,” “If I am infected with HPV, it would significantly affect my life,” “I think HPV-related cancer is a serious illness,” “If I got HPV-related cancer, it would be serious,” and “If I got HPV-related cancer, it would significantly affect my life.” These items utilized a 5-point Likert scale (1 = “strongly disagree” to 5 = “strongly agree”).

### Perceived benefits

Perceived benefits were assessed with three items, adapted from Tatar et al. (73): “I think that the HPV vaccine is effective in helping to prevent diseases caused by HPV,” “I think that the HPV vaccine may be effective in helping to prevent the HPV virus,” and “I think that the HPV vaccine may be effective in helping to prevent HPV-related cancer.” These items utilized a 5-point Likert scale (1 = “strongly disagree” to 5 = “strongly agree”).

### Subjective norm

Subjective norm were assessed with four items, adapted from Britt and Englebert (74): “Most people who are important to me think I should get the HPV vaccination,” “My parents or family members think I should get the HPV vaccination,” “My friends think I should get the HPV vaccination,” and “People who are important to me influence my decision to have HPV vaccination.” These items utilized a 5-point Likert scale (1 = “strongly disagree” to 5 = “strongly agree”).

### Perceived behavioral control

Perceived behavioral control were assessed with five items, adapted from De Perio et al. (75): “It is my decision whether to get the HPV vaccination,” “I am confident I could get the HPV vaccination if I wanted,” “I do have the time to get the HPV vaccination,” “I do have the money to get the HPV vaccination,” and “Getting the HPV vaccination does not require a lot of effort on my part.” These items utilized a 5-point Likert scale (1 = “strongly disagree” to 5 = “strongly agree”).

### Attitude

Attitude was measured by four items, adapted from Xiao (70): “For me, getting the HPV vaccine would be useful,” “For me, getting the HPV vaccine would be valuable,” “For me, getting the HPV vaccine would be important,” and “For me, getting the HPV vaccine would be effective.” These items utilized a 7-point Likert scale (1 = “strongly disagree” to 7 = “strongly agree”).

### Behavioral intention

Behavioral intention were assessed with five items, adapted from Gerend and Shepherd (33): “I will try to get more information about HPV vaccination,” “I will consider getting vaccinated for HPV,” “I will try to get vaccinated against HPV,” “I will actually get the HPV vaccination,” and “If the doctor provides me with the HPV vaccine, I will get vaccinated.” These items utilized a 7-point Likert scale (1 = “strongly disagree” to 7 = “strongly agree”).

### Data analysis

Partial Least Squares Structural Equation Modeling (PLS-SEM) has advantages in dealing with model complexity, which is more advantageous than covariance-based structural equation modeling. (76). Given the complexity and prediction-oriented nature of the conceptual model in this study, PLS-SEM was deemed the appropriate analytical technique (77, 78). The conceptual model in this study comprises seven latent variables, with six independent variables, one mediator, and one dependent variable, surpassing the mean in terms of complexity and the number of paths and variables, as noted by Hair et al. (76). Akter et al. (79) further supports the superiority of PLS-SEM for analyzing multiple regressions involving numerous independent and intervening variables within a single analytical framework.

Considering the intricate nature of this study's model, SmartPLS 4 (v 4.1.0.3) software was employed to assess the measurement and structural model. In line with Hair et al. (77, 80), evaluating of the reflective measurement model involves testing indicator loadings, internal consistency reliability, convergent validity, and discriminant validity. The subsequent phase involves the structural model assessment upon achieving satisfactory results in the measurement model evaluation. The standard criteria for structural model assessment encompass the Variance Inflated Factor, coefficient of determination (*R*^2^), effect sizes (*f*^2^), predictive relevance (*Q*^2^), and the significance of the path coefficients, ensuring a comprehensive analysis of the model's predictive and explanatory power.

## Results

### Measurement model assessment

In accordance with the recommendations of Hair et al. (77, 80), the measurement model was evaluated to establish reliability and convergent validity by examining Cronbach's α (>0.7), factor loadings (>0.5), composite reliability (>0.7), and average variance extracted (>0.5). As shown in Table 2, the results indicate that Cronbach's α values range from 0.830 to 0.961. The factor loadings are within the interval of 0.573 to 0.942. The composite reliability values are reasonable, falling between 0.881 and 0.970. Additionally, the average variance extracted values range from 0.556 to 0.864.

**Table 2:** Result of reliability and convergent validity.

**Constructs**	**Measurement items**	**Factor loading**	**Cronbach's Alpha**	**Composite reliability**	**Average variance extracted**
Behavioral intention (BI)	BI1	0.919	0.961	0.970	0.864
	BI2	0.939			
	BI3	0.942			
	BI4	0.926			
	BI5	0.922			
Attitude (ATT)	ATT1	0.787	0.845	0.896	0.683
	ATT2	0.859			
	ATT3	0.862			
	ATT4	0.794			
Subjective norm (SN)	SN1	0.796	0.867	0.907	0.710
	SN2	0.843			
	SN3	0.865			
	SN4	0.863			
Perceived behavioral control (PBC)	PBC1	0.794	0.875	0.909	0.668
	PBC2	0.892			
	PBC3	0.759			
	PBC4	0.842			
	PBC5	0.793			
Perceived susceptibility (PSU)	PSU1	0.849	0.894	0.903	0.613
	PSU2	0.846			
	PSU3	0.913			
	PSU4	0.725			
	PSU5	0.746			
	PSU6	0.573			
Perceived severity (PSE)	PSE1	0.812	0.842	0.881	0.556
	PSE2	0.839			
	PSE3	0.855			
	PSE4	0.678			
	PSE5	0.610			
	PSE6	0.643			
Perceived benefits (PB)	PB1	0.837	0.830	0.892	0.734
	PB2	0.880			
	PB3	0.852			

Discriminant validity was evaluated using the conservative heterotrait—monotrait (HTMT) ratio approach recommended by Henseler et al. (81), Table 3 confirms all HTMT values below the 0.85 threshold, indicating no discriminant validity issues among the constructs. The results demonstrated that the measurement model satisfies the reliability and validity in this study.

**Table 3:** HTMT ratio analysis.

**Construct**	**ATT**	**BI**	**PB**	**PBC**	**PSE**	**PSU**	**SN**
ATT							
BI	0.652						
PB	0.301	0.380					
PBC	0.460	0.673	0.280				
PSE	0.571	0.396	0.232	0.262			
PSU	0.139	0.158	0.102	0.089	0.085		
SN	0.234	0.234	0.248	0.189	0.129	0.073	

### Structural model assessment

Table 4 presents the values of the determination coefficient (*R*^2^) and predictive relevance (*Q*^2^). Attitude variance is 29.5% explained by perceived susceptibility, severity, and benefits, while 53.2% of behavioral intention variance is attributable to attitude, subjective norms, and perceived behavioral control. *R*^2^ values indicated that all external variables have a satisfactory effect on internal dependent variables (77, 78). Moreover, *Q*^2^ values for a specific endogenous construct should be greater than zero to indicate good predictive relevance (81). As shown in Table 4, all Q^2^ values are greater than 0 (attitude = 0.200, behavioral intention = 0.457), confirming that the PLS-SEM model exhibits good predictive power.

**Table 4:** R^2^ and Q^2^.

**Endogenous variables**	***R* ^2^**	***Q* ^2^**
Attitude	0.295	0.200
Behavioral intention	0.532	0.457

**Figure 2:**
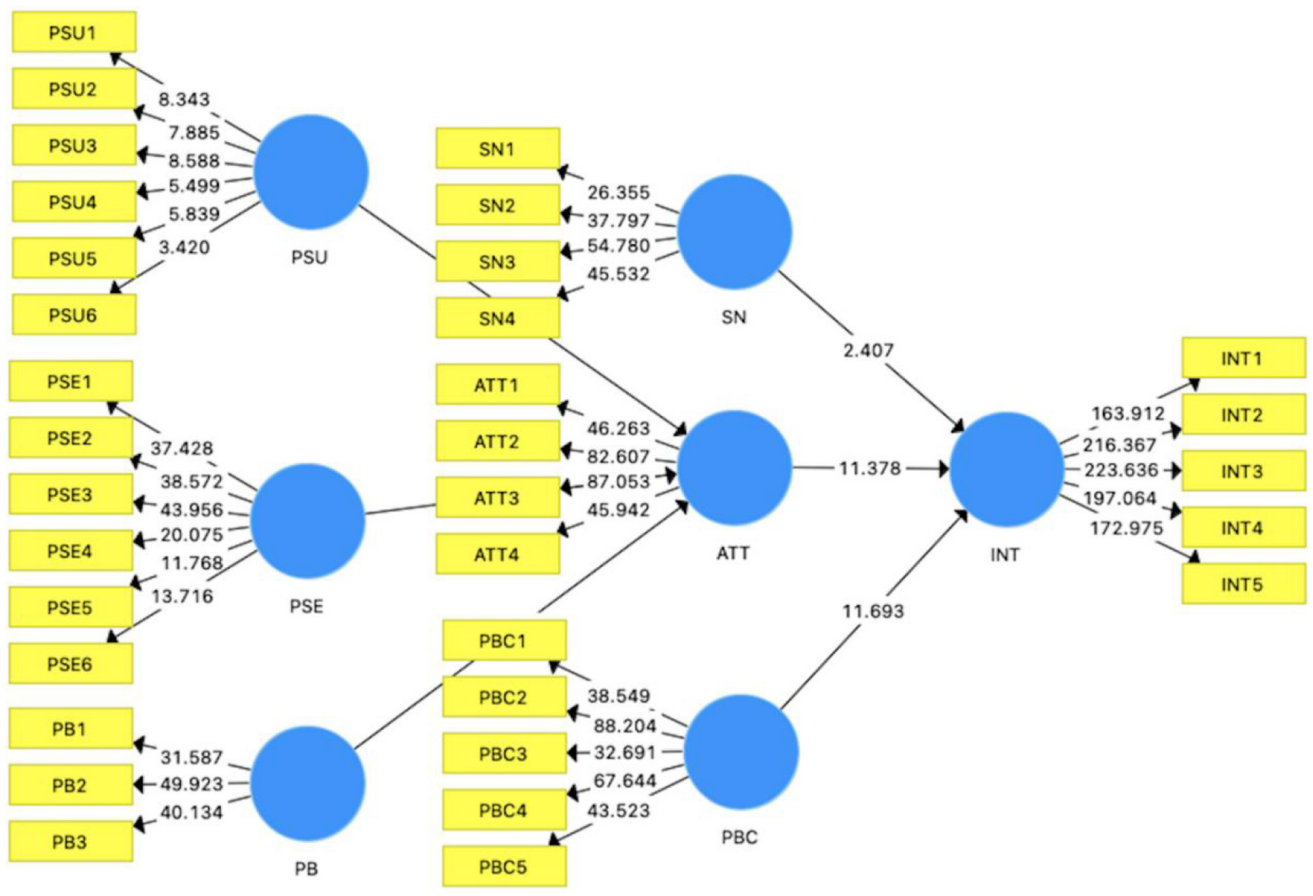
Results of path coefficients.

Following Hair et al. (77, 80), all hypotheses were assessed using 5000 bootstrap samples with one-tailed testing. The PLS-bootstrapping results shown in Figure 2 and Table 5 presents that perceived susceptibility (β = 0.109, *t* = 2.658, *p* < 0.05), perceived severity (β = 0.456, *t* = 13.323, *p* < 0.05), and perceived benefits (β = 0.175, *t* = 5.413, *p* < 0.05) have significant positive effects on attitude toward the HPV vaccine. These findings support hypotheses H1, H2, and H3. Besides, subjective norm (β = 0.070, *t* = 2.407, *p* < 0.05), attitude (β = 0.397, *t* = 11.378, *p* < 0.05), and perceived behavioral control (β = 0.454, *t* = 11.693, *p* < 0.05) have significant positive effects on the intention to receive HPV vaccination. Thus, hypotheses H4, H5, and H6 are supported. Besides, the VIF was employed to assess multicollinearity among items. The VIF values ranged from 1.019 to 1.215, indicating no multicollinearity issue.

**Table 5:** Path coefficients.

**Paths**	**Beta value**	**Standard deviation**	***T* values**	***P* values**	***f^2^***	**CILL**	**CILU**	**VIF**	**Decision**
PSU → ATT	0.109	0.041	2.658	0.004	0.017	0.019	0.146	1.019	Support
PSE → ATT	0.456	0.034	13.323	0.000	0.282	0.396	0.509	1.055	Support
PB → ATT	0.175	0.032	5.413	0.000	0.041	0.124	0.230	1.060	Support
SN → BI	0.070	0.029	2.407	0.008	0.010	0.020	0.117	1.054	Support
ATT → BI	0.397	0.035	11.378	0.000	0.279	0.339	0.453	1.215	Support
PBC → BI	0.454	0.039	11.693	0.000	0.371	0.389	0.517	1.197	Support

CILL, confidence interval lower limit; CIUL, confidence interval upper limit.

### Mediation effect

In this study, the attitude was treated as a mediating effect. The mediation effect was assessed using the bootstrap method proposed by Preacher and Hayes (82), which is endorsed by Hair et al. (80) as a suitable approach for PLS-SEM. As shown in Table 6, Attitude serves as a positive mediator in the relationship between perceived susceptibility (β = 0.043, *t*-value = 2.598, *p* < 0.05), perceived severity (β = 0.181, *t*-value = 8.114, *p* < 0.05), perceived benefits (β = 0.070, *t*-value = 4.918, *p* < 0.05), and intention to receive HPV vaccination. Therefore, hypotheses H7, H8, and H9 are supported.

**Table 6:** Results of the mediation analysis.

**Paths**	**Beta value**	**Standard deviation**	***T* values**	***P* values**	**CILL**	**CILU**	**Decision**
PSU → ATT → BI	0.043	0.017	2.598	0.005	0.013	0.060	Support
PSE → ATT → BI	0.181	0.022	8.114	0.000	0.146	0.220	Support
PB → ATT → BI	0.070	0.014	4.918	0.000	0.048	0.095	Support

CILL, confidence interval lower limit; CIUL, confidence interval upper limit.

## Discussion

This study examines young adults' HPV vaccine perceptions and vaccination intention, targeting a high-risk demographic within catch-up immunization age. The data analysis outcomes provide empirical support for all the hypotheses examined in this research. Key findings indicate perceived susceptibility and severity significantly increase HPV vaccination intention among young adults. This aligns with prior research, such as Marlow et al. (17) and Rose et al. (83), who reported that higher perceived susceptibility and perceived severity correlate with a higher intention to receive HPV vaccination. This outcome is anticipated as individuals' assessment of health risks or threats can generate uncertainty, which may either encourage or discourage them from taking preventive measures. The findings support the notion that “prevention is better than cure.” If young adults believe that being susceptible to HPV infection could lead to serious consequences, they are more likely to prioritize vaccination as a preventive measure. Hence, it is crucial to design campaigns that specifically target these perceptions. Social media platforms, for example, can be effectively utilized to disseminate evidence-based information. This information should clearly highlight the risks of HPV infection and the severity of its related health consequences.

Perceived benefits of HPV vaccination have a positive impact on young adults' intention to receive HPV vaccination. This result consistent with several previous studies, which have consistently indicated that understanding the benefits of HPV vaccination positively affects the intention to vaccination, particularly among females (17, 18). The positive perception of the benefits of HPV vaccination stems from several factors. Firstly, HPV vaccination campaigns and education initiatives have played a key role in highlighting the long-term health benefits of vaccination, including the prevention of cervical cancer and other diseases caused by HPV. These campaigns spread information about the effectiveness and safety of vaccines, helping to eliminate misunderstandings and alleviate concerns that individuals may be prevented from seeking vaccination. Besides, the social and cultural context can also influence the perceived benefits of HPV vaccination. In a society that values health awareness and preventive medicine, individuals are more likely to view vaccination as a beneficial and responsible health choice. It is reinforced by the endorsement of HPV vaccination by healthcare professionals and public health organizations, which adds credibility and trust to the perceived benefits.

The concept of subjective norm, which encompasses the societal expectations and opinions of valued individuals such as family, friends, and peers, influences health behaviors. The normative influence is particularly pronounced in collectivistic societies, where there is a strong inclination to conform to social norms and to be integrated within the community (84). In such contexts, the subjective norm has been identified to have a significant positive correlation with young adults' intentions to receive HPV vaccination. It aligns with the empirical findings of Li and Li (85), who observed a positive relationship between subjective norms and the intention of young females to receive HPV vaccination in China. Besides, the role of healthcare professionals, particularly doctors, in shaping vaccination behavior cannot be understated. Studies have shown that medical advice regarding HPV vaccination is significantly associated with the likelihood of females receiving the vaccine (51, 86). Although young adults over the age of 18 are not legally required to seek parental consent for vaccination, parental approval and the desire to align with parental expectations continue to play a pivotal role in vaccination decisions. It underscores the fact that in a collectivistic culture like China's, people are more inclined to follow behaviors that are considered appropriate by the majority (87). The findings of this study, therefore, highlight the importance of positive reinforcement from significant others in promoting the intention to receive HPV vaccination. It suggests that communication strategies that emphasize the endorsement and support of the HPV vaccine by influential figures in an individual's life can be instrumental in encouraging vaccination uptake.

The empirical results reveal that attitude is positively correlated with young adults' intention to receive HPV vaccination. This positive link between attitude and intention shows that when young adults hold favorable views toward HPV vaccination, they are more inclined to get vaccinated. The consistency of this finding with previous studies (49, 50) indicates that the relationship between attitude and health behavior has a robust pattern. It emphasizes the importance of shaping public attitudes and addressing concerns that may affect individuals' attitudes toward vaccination. In addition, the findings emphasize the need for public health campaigns and educational interventions to improve attitudes toward HPV vaccination. These efforts can foster a more positive attitude among young adults by providing clear evidence-based information on HPV vaccination and eliminating misunderstandings. This, in turn, will increase the intention to receive the vaccine, and ultimately promote higher vaccination rates and improve public health outcomes.

As anticipated, the empirical results revealed a positive association between perceived behavioral control and young adults' intentions to receive HPV vaccination. Specifically, the stronger the perceived behavioral control among young adults, the more pronounced their intention to get vaccinated. This outcome is congruent with previous studies (56, 85, 88). In this study, perceived behavioral control (*f*^2^ = 0.371) exerts a significant influence on the intention to receive HPV vaccination, underscoring its significance role in young adults' decision-making process. This belief in control is predicated on a combination of external and internal factors, including opportunities, competencies, resources, information access, and self-determination (52, 89). The perception of greater resources and fewer barriers enhances individuals' perceived behavioral control, which in turn enhances their intention to to receive HPV vaccination. Consequently, future interventions should not only strengthen the knowledge of HPV vaccination, but also focus on enhancing self-efficacy and perceived control of HPV vaccination. For example, interventions could provide practical resources and information about vaccination sites, costs and appointment procedures through accessible platforms such as social media or community health centers. These interventions can empower the confidence and autonomy of young adults and enable them to make informed decisions about their health, which may increase the HPV vaccination rate.

The results revealed the mediating role of attitude toward HPV vaccines between perceived sensitivity, perceived severity, perceived benefit and intention to receive the intention. This mediating effect suggests that the individual's overall attitude is a key mechanism that links perception with behavioral intention. The consistency of these findings with previous studies (68, 90, 91) reinforces the validity of the mediating effect of attitude in various research contexts. The results confirmed the mediating role of attitude in the decision-making process of HPV vaccination among young adults. Public health initiatives can be more effectively designed to promote HPV vaccination uptake. Specifically, regarding perceptions of HPV susceptibility and severity, public health initiatives can utilize social media to disseminate evidence-based information about HPV risks and vaccination benefits. Additionally, collaborating with healthcare professionals and trusted community figures ensures the dissemination of accurate and reliable information, which is vital for cultivating positive attitudes toward HPV vaccination.

## Conclusion

This study, based on an integrated model of the HBM and TPB, examined the factors influencing young adults' intention to receive the HPV vaccination, with a particular focus on the mediating role of attitude. PLS-SEM was utilized for data analysis. The results indicated that perceived susceptibility, perceived severity, and perceived benefits significantly and positively influenced attitudes toward the HPV vaccine. Subjective norms, attitude, and perceived behavioral control positively affected the intention to receive the HPV vaccination. Attitude played a significant mediating role between perceived susceptibility, perceived severity, perceived benefits, and intention to receive HPV vaccination. The study underscores the importance of implementing targeted initiatives to enhance young adults' attitudes toward HPV vaccination, which can increase vaccination rates and promote public health. Specifically, it is essential to design promotional campaigns through social media that target susceptibility and severity, using evidence-based information to highlight the risks of HPV and the benefits of vaccination. Additionally, Supportive communication from influential figures, such as family, friends, and healthcare professionals, significantly enhances HPV vaccination intention, Moreover, providing clear and practical information about HPV vaccination to improve perceived behavioral control can help young people make informed decisions. When these strategies are combined with accurate information and tailored to the cultural context, they can effectively shape positive attitudes and increase the intention to receive the HPV vaccination.

## Limitations and future work

This study explored the factors influencing the intention of young adults to receive HPV vaccination in China. However, there are still some limitations to be addressed in future research. First, the cross-section design limits causal inference. Future research should adopt a longitudinal approach to observe the evolution of HPV vaccination intention over time, so as to provide a more comprehensive understanding of the factors affecting vaccination behavior. Secondly, the current study focuses on constructs from the TPB and the HBM. Future research could consider incorporating additional influential factors. For instance, cultural values, social norms, and the potential stigmatization associated with the decision to receive HPV vaccination. Thirdly, the research is based on self-reported data, which may be affected by social desirability bias and recall errors. Future research should explore utilizing multiple data sources, such as medical records and administrative data. Fourthly, the convenience sampling method may limit the generalizability of findings due to potential selection bias. Future studies could employ stratified or probability sampling to enhance representativeness.

## Data Availability

The raw data supporting the conclusions of this article will be made available by the authors, without undue reservation.
